# Evaluation of Different Recruitment Methods: Longitudinal, Web-Based, Pan-European Physical Activity Through Sustainable Transport Approaches (PASTA) Project

**DOI:** 10.2196/11492

**Published:** 2019-05-06

**Authors:** Mailin Gaupp-Berghausen, Elisabeth Raser, Esther Anaya-Boig, Ione Avila-Palencia, Audrey de Nazelle, Evi Dons, Helen Franzen, Regine Gerike, Thomas Götschi, Francesco Iacorossi, Reinhard Hössinger, Mark Nieuwenhuijsen, David Rojas-Rueda, Julian Sanchez, Emilia Smeds, Manja Deforth, Arnout Standaert, Erik Stigell, Tom Cole-Hunter, Luc Int Panis

**Affiliations:** 1 Institute for Transport Studies University of Natural Resources and Life Sciences, Vienna Vienna Austria; 2 Centre for Environmental Policy Imperial College London London United Kingdom; 3 ISGlobal Barcelona Spain; 4 Universitat Pompeu Fabra Barcelona Spain; 5 Centro de Investigación Biomédica en Red de Epidemiología y Salud Pública Madrid Spain; 6 Centre for Environmental Sciences Hasselt University Hasselt Belgium; 7 Flemish Institute for Technological Research (VITO) Mol Belgium; 8 ICLEI European Secretariat GmbH Freiburg Germany; 9 Chair of Integrated Transport Planning and Traffic Engineering Technische Universität Dresden Dresden Germany; 10 Physical Activity and Health Unit, Epidemiology Biostatistics and Prevention Institute University of Zurich Zurich Switzerland; 11 Agenzia Roma Servizi per la Mobilità Rome Italy; 12 London Borough of Newham London United Kingdom; 13 The London School of Economics and Political Science London United Kingdom; 14 Department of Science, Technology, Engineering and Public Policy University College London London United Kingdom; 15 Trivector Traffic AB Stockholm Sweden; 16 International Laboratory for Air Quality and Health Institute of Health and Biomedical Innovation Queensland University of Technology Brisbane Australia; 17 Centre for Air Pollution, Energy and Health Research Sydney Australia; 18 Transportation Research Institute (IMOB) Hasselt University Diepenbeek Belgium

**Keywords:** longitudinal survey, multicentral, Web-based survey, opportunistic sampling, recruitment, Web-based questionnaire, mobile phone

## Abstract

**Background:**

Sufficient sample size and minimal sample bias are core requirements for empirical data analyses. Combining opportunistic recruitment with a Web-based survey and data-collection platform yields new benefits over traditional recruitment approaches.

**Objective:**

This paper aims to report the success of different recruitment methods and obtain data on participants’ characteristics, participation behavior, recruitment rates, and representativeness of the sample.

**Methods:**

A longitudinal, Web-based survey was implemented as part of the European PASTA (Physical Activity through Sustainable Transport Approaches) project, between November 2014 and December 2016. During this period, participants were recruited from 7 European cities on a rolling basis. A standardized guide on recruitment strategy was developed for all cities, to reach a sufficient number of adult participants. To make use of the strengths and minimize weakness, a combination of different opportunistic recruitment methods was applied. In addition, the random sampling approach was applied in the city of Örebro. To reduce the attrition rate and improve real-time monitoring, the Web-based platform featured a participant’s and a researchers’ user interface and dashboard.

**Results:**

Overall, 10,691 participants were recruited; most people found out about the survey through their workplace or employer (2300/10691, 21.51%), outreach promotion (2219/10691, 20.76%), and social media (1859/10691, 17.39%). The average number of questionnaires filled in per participant varied significantly between the cities (*P*<.001), with the highest number in Zurich (11.0, SE 0.33) and the lowest in Örebro (4.8, SE 0.17). Collaboration with local organizations, the use of Facebook and mailing lists, and direct street recruitment were the most effective approaches in reaching a high share of participants (*P*<.001). Considering the invested working hours, Facebook was one of the most time-efficient methods. Compared with the cities’ census data, the composition of study participants was broadly representative in terms of gender distribution; however, the study included younger and better-educated participants.

**Conclusions:**

We observed that offering a mixed recruitment approach was highly effective in achieving a high participation rate. The highest attrition rate and the lowest average number of questionnaires filled in per participant were observed in Örebro, which also recruited participants through random sampling. These findings suggest that people who are more interested in the topic are more willing to participate and stay in a survey than those who are selected randomly and may not have a strong connection to the research topic. Although direct face-to-face contacts were very effective with respect to the number of recruited participants, recruiting people through social media was not only effective but also very time efficient. The collected data are based on one of the largest recruited longitudinal samples with a common recruitment strategy in different European cities.

## Introduction

Recruiting participants has become increasingly challenging in the face of privacy concerns, an increasing number of surveys, expectations of rewards for survey completion, and necessary effort to achieve an unbiased representative sample [1]. Sufficient sample sizes and minimal sample bias are core requirements for empirical data collection in order to properly obtain answers to research questions [2]. Meeting these requirements is especially challenging for surveys with high response burdens such as longitudinal studies [3]. Traditional recruitment methods, such as mailed invitations based on samples drawn from population registries, are costly and increasingly yield sample biases due to declining response rates and selectivity effects [4-7], for example, because of an increase in the exclusive use of mobile phones or email rather than traditional mail or landline phone. Opportunistic approaches, such as recruitment through social media, promise cost savings [4,7-12] and a better coverage of person groups that are hard to reach with the traditional recruitment methods [13], like parents of adolescents [8], adolescents themselves [10], people with special conditions [14,15], smokers [16], low-income people [17], or people with a disproportionate risk for poor health outcomes [18]. Social media, like Facebook and Twitter, can potentially have a strong snowballing effect [19], given their intensive use and continuing growth (around 1.94 billion monthly active Facebook users worldwide [20]) and their features that allow information to be shared very easily among networks [19]; therefore, they are able to reach a large number of people in a very short time. Combining opportunistic recruitment with a Web-based survey and data-collection platform comprises additional benefits such as real-time monitoring of recruitment progress and enabling ongoing optimization of recruitment activities [4]. Poor response rates can further be improved by including rewards for participation such as financial compensations [21]. One major drawback of the opportunistic recruitment methods is the concern of sample bias, as the population sampled does not necessarily represent each group of the total population equally well [10]. Specific types of social media might, for example, be preferably used by younger people [3,7,11] and not by the elderly. In this case, the elderly have little chance to be sampled at all. Other, more traditional methods, such as flyers, involve problems of respondents not having a direct and convenient access to the survey and may result in a smaller recruitment rate [22].

The PASTA (Physical Activity through Sustainable Transport Approaches) Project [23,24] used a combination of different opportunistic recruitment methods to utilize strengths and minimize weaknesses. The project study collected data in a longitudinal, Web-based survey with a cohort design to study the effects of active mobility (like walking and cycling) on the overall physical activity and health, crash risks, and exposure to traffic-related air pollution. Data collection was performed in seven European cities: Antwerp, Barcelona, London, Örebro, Rome, Vienna, and Zurich. The target population for the project survey was the entire adult population in each of the seven case study cities, with the aim of oversampling participants who use a bicycle for their daily trips.

The objective of this paper was to report the success of these different methods with regard to obtaining participants’ characteristics, participation behavior, recruitment rates, and representativeness of the sample. More specifically, we aimed to (1) describe participant characteristics in the seven European cities in terms of the number of recruited people, gender, age, education level, and employment status; (2) show how participants found out about the survey; (3) illustrate participation behavior by reporting on the number of filled-in questionnaires, attrition, and withdrawal rate; (4) present the effectiveness (the number of predicted participants) and time efficiency of different recruitment approaches; and (5) compare our sample with the general population. Finally, we present our conclusions for the use of recruitment studies in future research on comparable topics.

## Methods

### Approach

**Figure 1:**
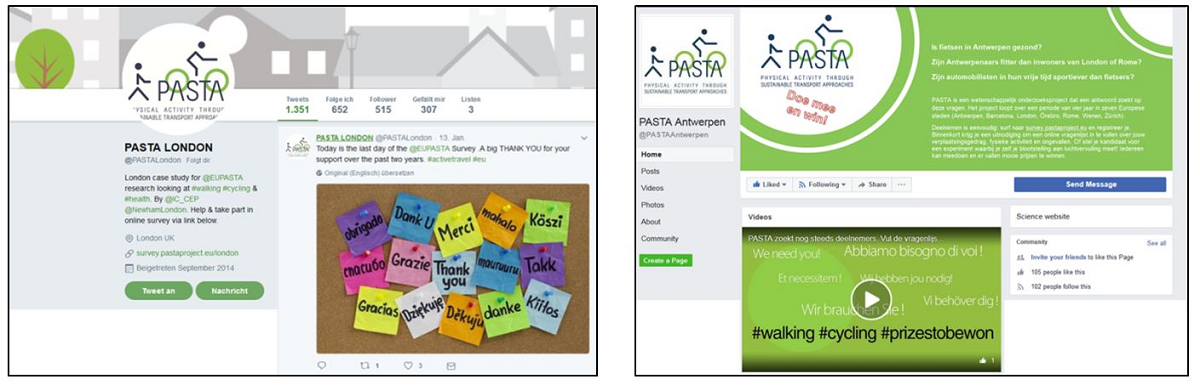
Cities’ own Twitter and Facebook accounts to recruit and inform people. Left: A Twitter page from London (Source: PASTA consortium, 2016, Project profile [Twitter]). Right: A Facebook page from Antwerp (Source: PASTA consortium, 2016, Project profile [Facebook]).

The PASTA project used a longitudinal, Web-based survey between November 2014 and December 2016. After the first questionnaire, which collected baseline information, participants received a follow-up questionnaire every 13 days to collect prospective data on travel behavior, levels of physical activity, and traffic safety incidents [23]. During this period, participants in the seven European cities were recruited on a rolling basis by using different opportunistic approaches. To reach a sufficient number of adult participants, a standardized guide on recruitment strategy was developed for all cities. A common recruitment strategy was important to achieve a similar number of participants and ensure more evenly distributed participation across transport modes and social groups in each city. The strategy for all cities included press releases and editorials; common promotional materials following the same visual identity guidelines; direct targeting of local stakeholders and community groups to distribute survey information through their communication channels (like newsletters, intranet, and webpages); extensive use of social media (each city had its own Facebook and Twitter pages where the link to the survey was regularly posted; Figure 1); and incentivizing for participation (ie, participants entered into a prize draw if they completed a questionnaire). The chance of participants winning increased with each additional completed questionnaire, except for participants in Sweden (Örebro) where the lottery was not allowed by law.

**Figure 2:**
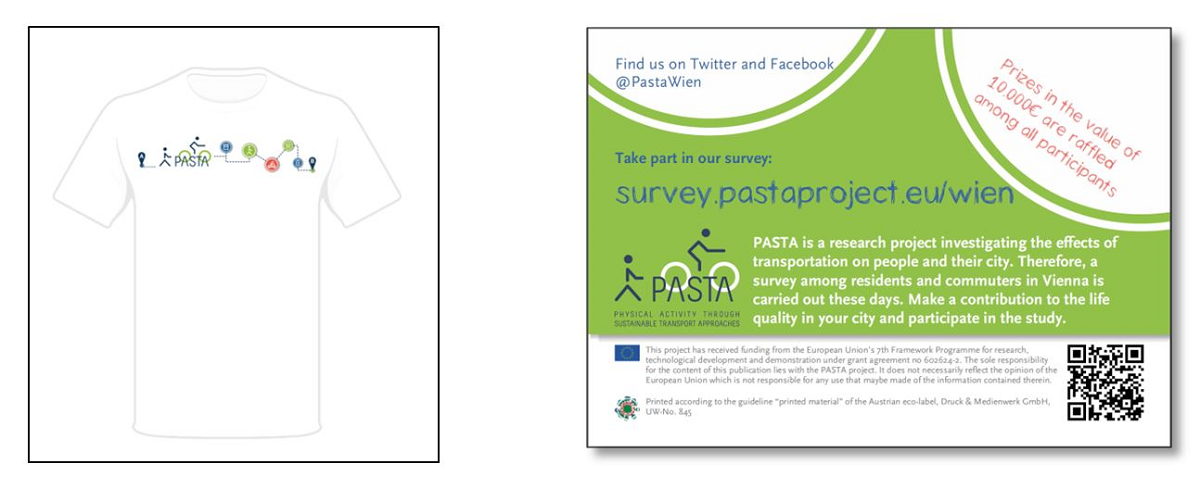
Recruitment material. Left: T-shirts worn by recruiters during outreach activities in Barcelona. Right: Registration postcard distributed in the city of Vienna.

A professional designer created a visual identity to enable clear and easy recognition of the project. All recruitment materials produced under the project were designed in line with the visual identity, which resulted in the creation of a strong brand (Figure 2). The survey as well as different dissemination materials (eg, flyers) were developed first in English and then translated into the local languages (ie, Dutch, Spanish, Catalan, Swedish, Italian, Swizz German, and Austrian German). This guaranteed that the same recruitment materials with the same contents were used in each city. Within this framework, there was flexibility to enable local initiatives and targeted city-specific recruitment, such as promoting recruitment for the project at social and cultural events. Furthermore, the city of Örebro applied an additional random sampling approach by contacting people aged 18-74 years through mail or phone.

To ensure high-data quality, several measures were put in place to reduce attrition rates, such as a user-friendly and custom-made survey platform and the automatic sending of reminder emails. Furthermore, participants were able to log into the platform at any time. There, they received an overview of their personal completed and open questionnaires and were able to complete unfinished questionnaires. Furthermore, they were given the opportunity to withdraw themselves actively from the survey if they did not want to participate any longer. In addition to the participants’ user interface, the platform also featured a researchers’ user interface and dashboard for real-time monitoring of recruitment and survey data collection. A user engagement strategy was also developed, including regular contact with the respondents, project branding, regular posting on social media, and keeping the project website up-to-date.

Participants were asked in the baseline questionnaire how they found out about the survey. They were given a choice between several different options, ranging from word of mouth to large-scale advertising campaigns. This question was answered by all participants who finalized the baseline questionnaire. At the same time, all city partners kept records on their local recruitment activities to measure invested efforts, including date, category, description, and invested time for each applied recruitment activity. Different categories were classified as follows: collaboration with local administration or organization (eg, survey link on webpages, newsletters, and intranet); handout of flyers at specific locations or specific events; display of posters at specific locations; use of mailing lists; advertisement in Web-based media or print media; papers in Web-based media, print media, or magazines; oral presentations for recruitment purposes; radio or television spots; Facebook; Twitter; street recruitment; and use of random sampling.

Ethics approval was obtained from the local ethics committees in the countries where the work was conducted and sent to the European Commission before the start of the survey.

### Statistical Analysis

Standard descriptive statistics outlined overall participant characteristics and were stratified by city, gender, age, education level, and employment status. To assess participation behavior, we tested the number of filled-in questionnaires by different sociodemographic characteristics using the nonparametric Kruskal-Wallis rank-sum test. Each significant result (*P*<.05) was followed by a Dunn test to account for significant differences within a variable. The sociodemographic characteristics of the sample (age and gender) were compared with each city’s census data by applying the Pearson chi-square test and size effect calculations.

To estimate the effectiveness (number of participants who started the baseline survey) of different recruitment approaches, we developed a recruit-prediction model in the form of a nonlinear least squares model in cities that provided the most comprehensive and detailed information on their local recruitment activities (Antwerp, Barcelona, and Vienna). The prediction model is based on the assumption that each recruitment activity generates an effect on the number of participants, which resembles a density function of a log-normal distribution, that is, a steep increase and a flat decrease:

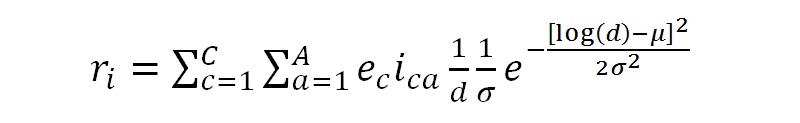



Where r_i_ denotes the predicted number of responses on day *i*; the index *c*=1, 2... *C* refers to different categories of recruitment activities (such as Facebook, Twitter, and flyer); the index *a*=1, 2... *A* refers to particular activities of category *C*; *e*_*c*_ is the intrinsic effectiveness of an activity of category *c*; *i*_*ca*_ is the intensity of a particular activity *a* of category *c*; *d* is the number of days elapsed since the start of the activity; *μ* and *σ* are the location and dispersion parameter of the log-normal density function, respectively. The curve characteristics of the density function were assumed to be the same for all recruitment categories: Only one set of parameters *μ* and *σ* was estimated for all categories. The intensity was assumed to vary as follows: each category has its intrinsic (baseline) effectiveness *e*_*c*_; within a given category, the intensity varies according to the invested effort *i*_*ca*_ (indicated by the reported number of working hours); and in some cases, with strong peaks of recruited people, which could unambiguously be assigned to particular recruitment activities with exceptional success, the intensity parameter was manually increased to capture the success of this activity adequately.

To estimate the time efficiency of different recruitment categories, we divided the number of predicted participants by the number of invested hours for each recruitment category. This was possible because the hours of work for project members to apply each activity (except for Facebook and Twitter posts) was recorded in detail. For social media activities, an average time value of 5 minutes per post was used. All statistical analyses were performed using R (version 3.4.0; The R Foundation for Statistical Computing, Vienna, Austria). Values given throughout the text are mean (SE).

## Results

### Sociodemographic Characteristics of Participants

A total of 10691 participants were recruited over a period of 27 months in the seven European cities, ranging from 1844 (17.25%) individuals in Rome to 1356 (12.68%) in Zurich. In all cities, except Rome, more women than men were recruited, with an average age of 41.9 (SE 0.19) years for men and 40.0 (SE 0.17) years for women. Most participants who stated their educational level were highly educated, with 6180 participants (of 8525, 72.49%) possessing a university degree and 2217 participants (of 8525, 26.01%) possessing a secondary education. In addition, 5993 participants (of 9892, 60.58%) were full-time employees, followed by 1662 (of 9892, 16.80%) part-time employees and 1361 (of 9892 13.76%) students; furthermore, 876 (of 9892, 8.86%) participants had home duties, were retired, or were unemployed (Table 1). Regarding nationality, 3560 participants (of 8586, 41.46%) were from Western Europe (predominantly in Antwerp, Vienna, and Zurich), 2840 (of 8586, 33.08%) were from Southern Europe (Barcelona and Rome), and 1858 (of 8586, 21.64%) were from Northern Europe (Örebro and London; according to the Geographic Regions listed by the United Nations).

**Table 1:** Respondent demographic information by recruitment city.

Characteristics			Antwerp	Barcelona	London	Örebro	Rome	Vienna	Zurich	Total
Participants in the sample, n (%)			1445 (13.52)	1727 (16.15)	1446 (13.53)	1401 (13.10)	1844 (17.25)	1472 (13.77)	1356 (12.68)	10,691 (100)
	**Gender, n (%)**									
		Male	689 (47.68)	706 (40.88)	600 (41.49)	530 (37.83)	1130 (61.28)	682 (46.33)	585 (43.14)	4922 (46.04)
		Female	756 (52.32)	1021 (59.12)	846 (58.51)	871 (62.17)	714 (38.72)	790 (53.67)	771 (56.86)	5769 (53.96)
	**Age in years, mean (SE)**									
		Overall	43.5 (0.32)	37.3 (0.30)	40.4 (0.34)	45.2 (0.40)	40.3 (0.26)	39.7 (0.35)	40.6 (0.34)	40.9 (0.13)
		Male	44.8 (0.48)	38.2 (0.48)	41.3 (0.54)	46.3 (0.67)	40.6 (0.35)	41.2 (0.52)	43.0 (0.53)	41.9 (0.19)
		Female	42.4 (0.43)	36.8 (0.38)	39.8 (0.44)	44.6 (0.50)	40.0 (0.41)	38.4 (0.46)	38.8 (0.44)	40.0 (0.17)
	**Education level, n (%)**		N=1245	N=1366	N=1033	N=1036	N=1548	N=1167	N=1130	N=8525
		University education	1044 (83.86)	1100 (80.53)	875 (84.70)	691 (66.70)	1015 (65.57)	756 (64.78)	699 (61.86)	6180 (72.49)
		Secondary education	191 (15.34)	249 (18.23)	151 (14.62)	319 (30.79)	530 (34.24)	371 (31.79)	406 (35.93)	2217 (26.01)
		Primary education	6 (0.48)	11 (0.81)	1 (0.10)	26 (2.51)	0 (0)	37 (3.17)	22 (1.95)	103 (1.21)
		No degree	4 (0.32)	6 (0.44)	6 (0.58)	0 (0)	3 (0.19)	3 (0.26)	3 (0.27)	25 (0.29)
	**Employment status, n (%)**		N=1378	N=1595	N=1300	N=1257	N=1680	N=1377	N=1305	N=9892
		Full-time	951 (69.01)	978 (61.32)	813 (62.54)	815 (64.84)	1151 (68.51)	639 (46.41)	646 (49.50)	5993 (60.58)
		Part-time	281 (20.39)	200 (12.54)	180 (13.85)	116 (9.23)	183 (10.89)	277 (20.12)	425 (32.58)	1662 (16.80)
		Student	31 (2.25)	285 (17.87)	156 (12.00)	142 (11.38)	278 (16.55)	302 (21.93)	167 (12.80)	1361 (13.76)
		Home duties, retired, or unemployed	115 (8.35)	132 (8.28)	151 (11.62)	184 (14.64)	68 (4.05)	159 (11.55)	67 (5.13)	876 (8.86)

### How Participants Found Out About the Survey

Table 2 shows that for all 10,691 participants, the three main sources of finding out about the survey were workplaces or employers (2300, 21.51%), outreach promotion (2219, 20.76%) such as presenting the project at different events or street recruitment, and social media (1859, 17.39%). The results varied across cities. In Antwerp, London, and Zurich, the highest share of respondents were reached through workplaces, whereas respondents in Rome were primarily reached through social media announcements and those in Barcelona, Örebro, and Vienna were reached through outreach promotion. In terms of gender, men were more likely to be recruited through outreach promotion (976/4922, 19.83%) or social media (942/4922, 19.14%), while women were most likely to be recruited through their workplace (1399/5769, 24.25%). Although participants aged 30-60 years could be best reached through their workplace, those aged 20-29 and >60 years were reached most often through outreach activities. In addition, students and participants without employment could best be reached through outreach activities. In case of respondents without a school-leaving qualification, recruitment through workplace or outreach activities was also the most successful approach.

**Table 2:** Participants’ responses to the question, “How did you find out about this survey?” during the baseline questionnaire.

Characteristics		Work	Word of mouth	Other organizations	Outreach activities	News	Social media^a^	Public notice	Random sampling	Other	Don’t know
Participants (N=10,691), n (%)		2300 (21.51)^b^	1219 (11.40)	1358 (12.70)	2219 (20.76^b^)	800 (7.48)	1859 (17.39^b^)	186 (1.74)	360 (3.38)	368 (3.44)	22 (0.21)
**Gender, n (%)**				
	Male (N=4922)	901 (18.31)	576 (11.70)	681 (13.84)	976 (19.83^b^)	416 (8.45)	942 (19.14^b^)	113 (2.30)	158 (3.21)	151 (3.07)	8 (0.16)
	Female (N=5769)	1399 (24.25^b^)	643 (11.15)	677 (11.73)	1243 (21.55)	384 (6.66)	917 (15.90)	73 (1.27)	202 (3.50)	217 (3.76)	14 (0.24)
**City, n (%)**				
	Antwerp (N=1445)	376 (26.02^b^)	195 (13.49)	289 (20.00)	132 (9.13)	98 (6.78)	311 (21.52)	13 (0.90)	N/A^c^	28 (1.94)	3 (0.21)
	Barcelona (N=1727)	199 (11.52)	369 (21.37)	113 (6.54)	665 (38.51^b^)	41 (2.37)	220 (12.74)	19 (1.10)	N/A^c^	95 (5.50)	6 (0.35)
	London (N=1446)	317 (21.92^b^)	146 (10.10)	282 (19.50)	198 (13.69)	110 (7.61)	243 (16.80)	22 (1.52)	N/A^c^	125 (8.64)	3 (0.21)
	Örebro (N=1401)	343 (24.48)	15 (1.07)	36 (2.57)	550 (39.26^b^)	51 (3.64)	33 (2.36)	9 (0.64)	360 (25.70)	0 (0)	4 (0.29)
	Rome (N=1844)	337 (18.28)	244 (13.23)	141 (7.65)	158 (8.57)	253 (13.72)	533 (28.90^b^)	90 (4.88)	N/A^c^	88 (4.77)	0 (0)
	Vienna (N=1472)	233 (15.83)	155 (10.53)	315 (21.40)	329 (22.35^b^)	109 (7.40)	275 (18.68)	27 (1.83)	N/A^c^	25 (1.70)	4 (0.27)
	Zurich (N=1356)	495 (36.50^b^)	95 (7.01)	182 (13.42)	187 (13.79)	138 (10.18)	244 (17.99)	6 (0.44)	N/A^c^	7 (0.52)	2 (0.15)
**Age (years), n (%)**				
	20-29 (N=2339)	378 (16.16)	334 (14.28)	239 (10.22)	618 (26.42^b^)	138 (5.90)	393 (16.80)	75 (3.21)	58 (2.48)	100 (4.28)	6 (0.26)
	30-39 (N=2339)	721 (23.84^b^)	415 (13.72)	358 (11.84)	528 (17.46)	197 (6.51)	590 (19.51)	37 (1.22)	80 (2.65)	96 (3.17)	2 (0.07)
	40-49 (N=2339)	560 (24.39^b^)	231 (10.06)	298 (12.98)	400 (17.42)	199 (8.67)	435 (18.95)	28 (1.22)	63 (2.74)	79 (3.44)	3 (0.13)
	50-59 (N=2339)	474 (24.73^b^)	151 (7.88)	302 (15.75)	383 (19.98)	145 (7.56)	295 (15.39)	28 (1.46)	63 (3.29)	69 (3.60)	7 (0.37)
	>60 (N=2339)	153 (15.09)	75 (7.40)	146 (14.40)	266 (26.23^b^)	109 (10.75)	132 (13.02)	13 (1.28)	96 (9.47)	20 (1.97)	4 (0.39)
**Education level, n (%)**				
	University education (N=2339)	1405 (22.70)^b^	784 (12.67)	781 (12.62)	1193 (19.28)	449 (7.25)	1086 (17.55)	89 (1.44)	187 (3.02)	208 (3.36)	7 (0.11)
	Secondary education (N=2339)	454 (20.48)^b^	212 (9.56)	288 (12.99)	465 (20.97)^b^	180 (8.12)	392 (17.68)	53 (2.39)	106 (4.78)	64 (2.89)	3 (0.14)
	Primary education (N=2339)	17 (16.50)	6 (5.83)	12 (11.65)	29 (28.16)^b^	14 (13.59)	13 (12.62)	0 (0)	9 (8.74)	3 (2.91)	0 (0)
	No degree (N=2339)	7 (28.00)^b^	3 (12.00)	2 (8.00)	6 (24.00)	0 (0)	5 (20.00)	0 (0)	0 (0)	2 (8.00)	0 (0)
**Employment status, n (%)**				
	Full-time (N=2339)	1633 (27.25^b^)	644 (10.75)	738 (12.31)	1042 (17.39)	443 (7.39)	1023 (17.07)	61 (1.02)	191 (3.19)	211 (3.52)	7 (0.12)
	Part-time (N=2339)	408 (24.55^b^)	170 (10.23)	252 (15.16)	307 (18.47)	124 (7.46)	301 (18.11)	17 (1.02)	38 (2.29)	43 (2.59)	2 (0.12)
	Student (N=2339)	123 (9.04)	201 (14.77)	145 (10.65)	413 (30.35^b^)	83 (6.10)	248 (18.22)	67 (4.92)	32 (2.35)	48 (3.53)	1 (0.07)
	Home duties, retired, or unemployed (N=2339)	17 (1.94)	110 (12.56)	137 (15.64)	266 (30.37^b^)	84 (9.59)	137 (15.64)	18 (2.05)	79 (9.02)	26 (2.97)	2 (0.23)

^a^Social media refers to Facebook and Twitter.

^b^These values refer to the highest share of participants reached through different recruitment activities.

^c^N/A: Not applicable.

### Participation Rates and Behavior

**Figure 3:**
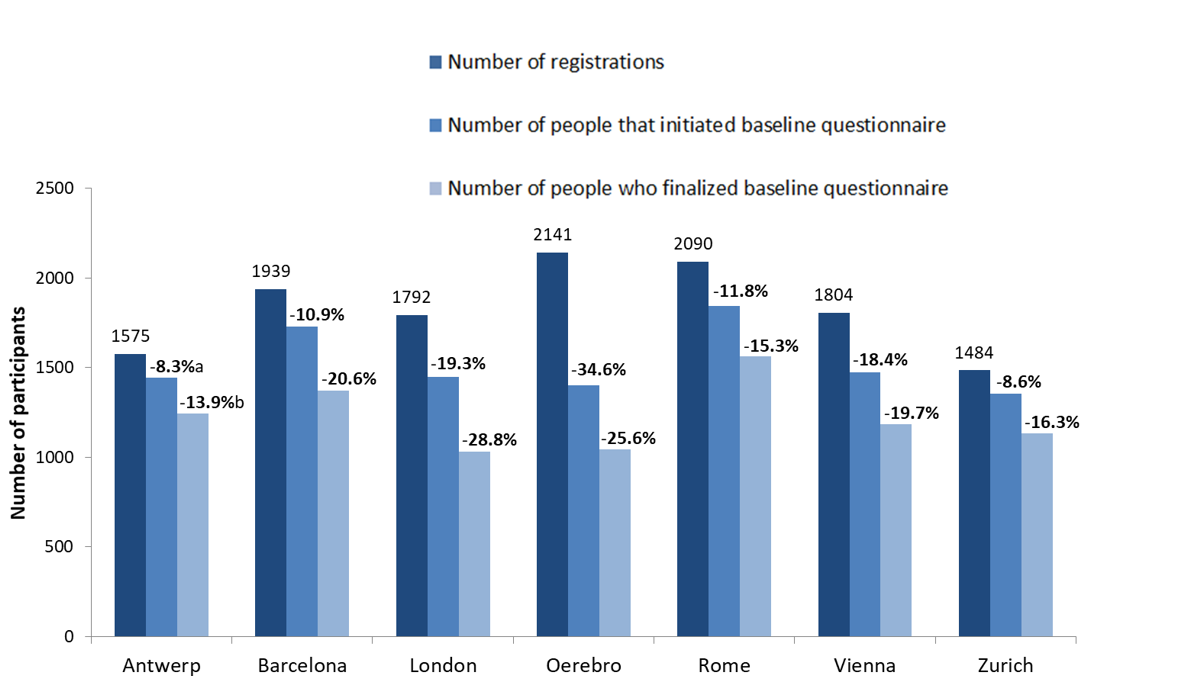
Number of participants who registered for the survey and who started and finalized the baseline questionnaire. (a) Attrition rate between the number of registrations and the number of started baseline questionnaires. (b) Attrition rate between the number of started and finalized baseline questionnaires.

A total of 12,825 people registered for the survey; however, 2134 never started the baseline questionnaire (attrition rate 16.64%). From the remaining 10,691 participants who started the baseline questionnaire, 8567 finalized it (additional attrition rate 19.87%). The attrition rates between people who registered, started, and finalized the baseline questionnaire varied across cities, with the lowest rates in Antwerp and the highest rates in Örebro and London (Figure 3).

The number of filled-in questionnaires per participant varied significantly across the cities (*P*<.001), with the highest number in Zurich (11.0, SE 0.33) and the lowest in Örebro (4.8, SE 0.17). In almost all cities, women filled in fewer questionnaires than men (7.7 [SE 0.1] vs 8.6 [SE 0.2]); furthermore, younger people or students tended to fill in fewer questionnaires than people aged 30-80 years or employees and people with home duties (*P*<.001). In addition, the way people were informed about the survey had a significant impact on the number of filled-in questionnaires (*P*<.001). Most questionnaires were filled in when people found out about the survey through other organizations (9.6, SE 0.3) or the news (9.5, SE 0.4; Table 3). In total, 12.17% (1301/10,691) participants withdrew from the survey, with the highest share in Örebro (311/1401, 22.20%) and the lowest share in Rome (82/1844, 4.45%).

**Table 3:** Number of filled-in questionnaires per participant by city, age groups, employment status, and mode through which participants found out about the survey.

Characteristics			Mean (SE)	Kruskal-Wallis test value (post hoc test: Dunn test)		*P* value
**City**				478.95		<.001^a^
	Antwerp		10.5 (0.32)^b^		
	Barcelona		8.2 (0.25)^c^		
	London		5.3 (0.20)^d^		
	Örebro		4.8 (0.17)^d^		
	Rome		7.4 (0.23)^e^		
	Vienna		10.0 (0.32)^f^		
	Zurich		11.0 (0.33)^b^		
**Age (years), n (%)**				91.679		<.001^a^
	<20		3.14 (0.39)^b^		
	20-29		6.85 (0.20)^c^		
	30-39		8.13 (0.19)^d^		
	40-49		8.80 (0.23)^d,e^		
	50-59		9.11 (0.26)^d,e^		
	≥60		8.33 (0.34)^d^		
**Employment status**				90.077		<.001^a^
	Full-time		8.9 (0.1)^b^		
	Part-time		9.2 (0.3)^b^		
	Student		6.7 (0.3)^d^		
	Home duties, retired, or unemployed		8.2 (0.4)^c^	
**Found out about survey through**				137.1		<.001
	Work		8.5 (0.2)^b^		
	Word of mouth		7.9 (0.3)^c^		
	Other organizations		9.6 (0.3)^b^		
	Outreach activities		6.9 (0.2)^d^		
	News		9.5 (0.4)^b^		
	Social media		8.6 (0.3)^b^		
	Public notice		5.5 (0.6)^e^		
	Random sampling		7.1 (0.5)^b,c^		
	Other		6.2 (0.2)^c,d^		

^a^Significant effect.

^b,c,d,e,f^Values with different letters differ significantly from each other (eg, mean values marked with “b” differ significantly from each mean value marked with “c,” “d,” “e,” or “f”).

**Table 4:** Effectiveness and time efficiency of different recruitment categories.

Recruitment categories		Effectiveness (n participants predicted)	*t* ^a^	*P* value ^a^	Time-efficiency (n participants predicted per working hour invested)
**Antwerp**				
	Facebook	402^b^	9.26	<.001^b^	41.2^b^
	Mailing lists	909^b^	80.48	<.001^b^	60.6^b^
	Collaborations with local administrations	9	1.17	.24	0.8
	Collaboration with local organizations	6	0.48	.63	0.2
	Flyer	19	2.18	.03^b^	1.1
	Poster	4	0.56	.58	1.0
	Radio	32	2.09	.04^b^	10.7
	Web-based advertisement	64^b^	8.67	<.001^b^	64.0^b^
**Barcelona**				
	Facebook	442^b^	10.08	<.001^b^	36.8^b^
	Mailing lists	66	5.55	<.001^b^	1.8
	Street recruitment	1002^b^	8.49	<.001^b^	0.9
	Print media	9	0.93	.35	3.0
**Vienna**				
	Facebook	534^b^	8.34	<.001^b^	33.0^b^
	Web-based media	39	4.08	<.001^b^	3.9
	Mailing list	21	2.32	.02^b^	2.3
	Collaboration with local administrations	149^b^	8.63	<.001^b^	2.3
	Collaboration with local organization	406^b^	7.27	<.001^b^	1.2
	Flyer	197	7.52	<.001^b^	2.1
	Poster	18	1.90	.06	3.6
	Street recruitment	86^b^	6.65	<.001^b^	2.7

^a^Columns 3 and 4 show the *t* and *P* values of the average parameter of a respective recruitment category; parameters of exceptionally successful activities (if available for a given category) are not shown (these parameters are always highly significant, eg, peak in Figure 4; the bottom graph). Residual SE: Antwerp, 4.552 (*df*=782); Barcelona, 3.028 (*df*=725); Vienna, 2.795 (*df*=719).

^b^Significant effect.

### Effectiveness and Efficiency of Different Recruitment Approaches

**Figure 4:**
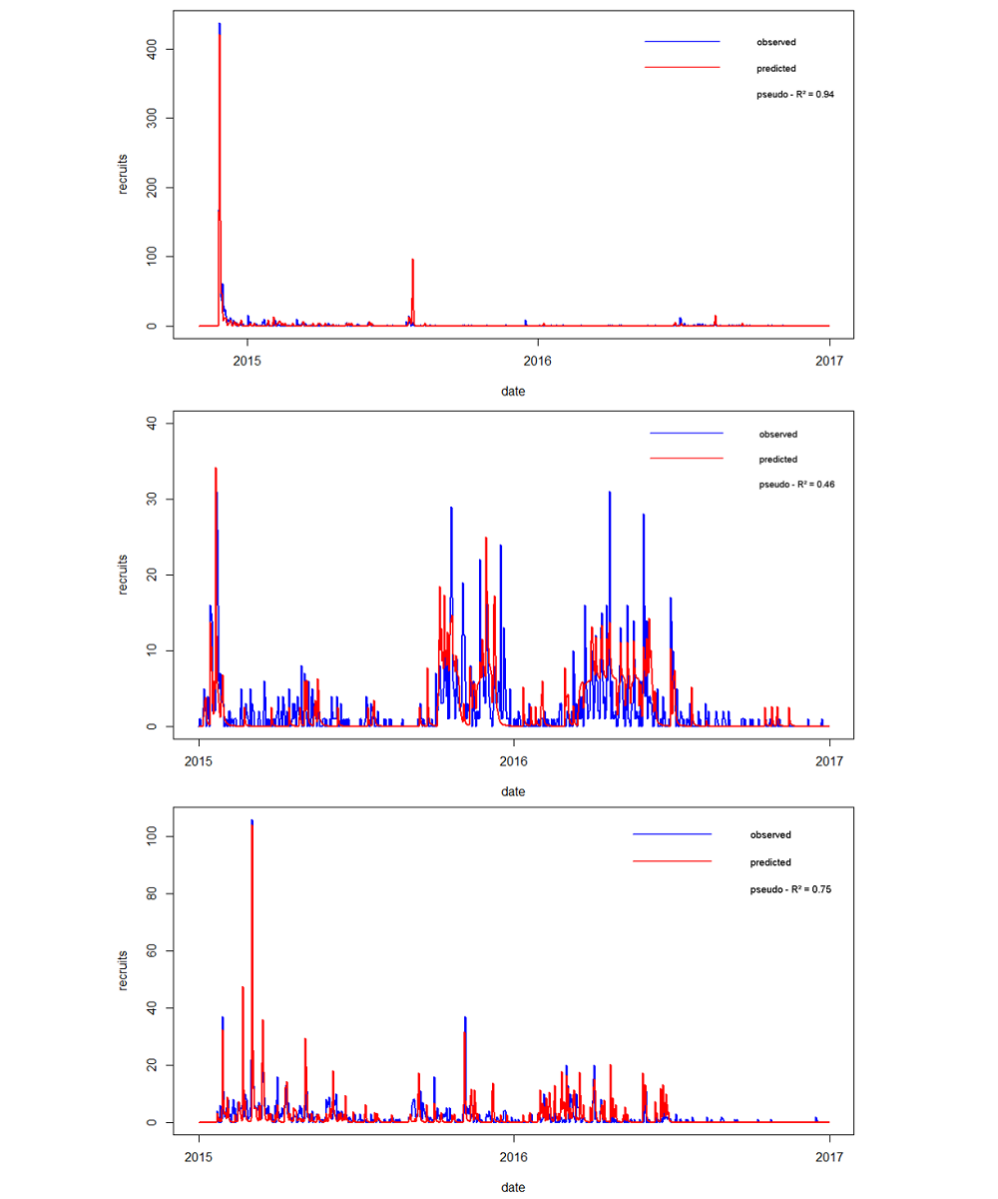
Number of recruited (observed) and predicted participants in Antwerp (top graph), Barcelona (middle graph), and Vienna (bottom graph; based on a nonlinear model).

Table 4 gives an overview of only effective recruitment categories, that is, only activities that were able to recruit participants according to the model. The categories in different cities were as follows: (1) Antwerp: Facebook, mailing lists, collaboration with local administrations and organization, use of flyers and posters, radio spots, and Web-based advertisement; (2) Barcelona: Facebook, mailing lists, street recruitment, and print media; and (3) Vienna: Facebook, Web-based media, mailing lists, collaboration with local administrations and organizations, use of flyers and posters, and street recruitment. One of the most effective approaches in all 3 cities was Facebook, with >400 predicted participants in Antwerp and Barcelona and >500 predicted participants in Vienna (*P*<.001**;** Table 4). In Antwerp, most people could be reached through different mailing lists (>900 participants) and in Barcelona, through a range of street recruitment activities (>1000 participants). In Vienna, especially, collaborations with local organizations (like the local bike sharing provider) were very effective in reaching a high share of predicted participants (eg, peak in Figure 4, the bottom graph). Considering the invested working hours, one of the most time-efficient categories in all 3 cities was Facebook, with >30 participants per invested working hour. Although mailing lists or Web-based advertisements were also effective in Antwerp, reaching approximately 60 participants per invested working hour, only 1 participant could be reached per working hour in Barcelona through street recruitment. In Figure 4, lines represent the sum of different recruitment activities. The nonlinear model does not provide an *R*^2^, because *R*^2^ is not defined for nonlinear models. We defined a pseudo *R*^2^ value, because the model is reasonably close to a linear model and the sample size is sufficiently large.

### Representativeness of the Sample

Compared with the cities’ census data, study participants in all cities, except Rome, were broadly representative in terms of gender distribution (*P*>.05). This was mainly the case if participants were informed about the survey by news, through word of mouth (friends, neighbors, or relatives) or social media (Multimedia Appendix 1). The main difference was that our recruited sample was on an average younger than the general population (high deviation from census data within the age class >60 years; Multimedia Appendix 2).

## Discussion

The main finding of this study was that Facebook was one of the most effective approaches in reaching a high share of participants. Considering the invested working hours, it was also one of the most time-efficient recruitment methods. Recruited participants were representative of the population in terms of gender but younger than the general population.

### Principal Results and Participation Behavior

The main source of information about the survey was workplaces or employers. Collaborations with different organizations who forwarded the survey information to their employees (eg, through their intranet or regular newsletters) was fundamental for raising awareness to drive recruitment. Outreach promotion by project members (eg, direct face-to-face recruitment at different events or on public places) and the use of social media channels (Facebook and Twitter) were the next most informative activities. This was especially the case in Rome, where social media was based on an account of the city council with a lot of followers.

One-third of people who registered for the survey did not complete the baseline questionnaire, with the highest attrition rate in Örebro and the lowest rate in Antwerp. In addition, Örebro had the lowest number of filled-in questionnaires per participant and the highest share of people who actively deregistered from the survey. One explanation may be the different approach adopted in Örebro, which also recruited participants through random sampling. The findings suggest that people who are more interested in the topic (in this case, active mobility research) are more willing to participate in a survey and more likely to stay in the study than those who are selected randomly and may not have a strong connection to the research topic [4-7]. Therefore, a (costly) random selection may eventually still lead to a biased sample that was to be avoid. However, poor response rates can also be improved by including rewards for participation [21]. The high attrition and deregistration rate in Örebro may also be (partly) caused by the fact that participation in Örebro was not rewarded.

### Comparative Effectiveness and Representativeness of Recruitment Methods

Although direct face-to-face contacts (eg, street recruitment) were very effective in terms of the number of recruited participants, recruiting people through social media (mainly Facebook) was not only effective but also very time efficient. Similar results were observed by others [4,10,11], who applied Web-based sampling in their research. By using the same medium (ie, internet), such sampling reduces the burden of participants because they can easily reach the survey by only clicking on a provided link. Regarding the near ubiquity of the internet, it has become easier for people to engage in surveys [25], as it can overcome barriers such as physical distance, transportation, and limited time [14]. Nevertheless, the effectiveness and time efficiency must be balanced with how representative the resulting sample is of the target population. Compared with the general population, study participants in almost all cities were broadly representative in terms of gender distribution. In particular, reaching people through news, word of mouth, or social media were the most successful options in recruiting a gender-balanced sample that represented a city’s population. There was, however, an age bias among the applied strategies compared with the city’s census data. Although the cities applied different strategies to include older people (eg, by visiting seniors who recently completed computer courses), most strategies attracted a higher proportion of younger people. Several studies, however, report successfully recruiting people who reflect the demographic spread of the general population using opportunistic sampling approaches [9,11,19,26]. People in our sample were further highly educated (6180/8525, 72.49% participants possessed a university degree), which is a common occurrence for survey research [9,27,28]. Nevertheless, this study found that targeting people without a school-leaving qualification through their workplaces or outreach activities was most promising; as such, some recruitment activities are better suited than others to attract hard-to-reach groups.

### Limitations and Strengths

Although this study represents a comprehensive examination of different recruitment approaches in a longitudinal, Europe-wide, Web-based survey, there were some limitations that could not be addressed by the research design. First, the sociodemographic characteristics of participants recruited by the discussed methods may be different for topics other than active mobility and the corresponding health aspects. The study population in our sample was highly educated and younger than the general population. This may be because our recruitment partly addressed cyclists, and the subject may hold particular interest to those with higher education. Second, participants needed to have access to the internet to participate in the Web-based survey, which could also explain the high proportion of young participants in this study. Finally, the sample was limited to the adult population. The strategies used may be effective in recruiting children or adolescents.

These limitations are offset by several strengths. First, we were able to recruit one of the largest longitudinal samples in different European cities with a common recruitment strategy. Second, we were able to shed new light on the effectiveness and time efficiency of different recruitment approaches. We now have a large and very detailed database on response behavior as per the recruitment method for seven different cities in Europe. We observed that offering a mixed recruitment approach was very effective in reaching a high participation rate. The resulting database can answer research questions and analyze the effects of active mobility on people’s health, crash risks, and exposure to traffic-related air pollution (eg, for cyclists) because of its size and composition. Thus, overall, the use of a mixed-methods approach has been successful.

## Appendix

Multimedia Appendix 1 Demographic characteristic (gender) by recruitment strategy of participants compared with each city's census.

Multimedia Appendix 2 Demographic characteristic (age groups) by recruitment strategy of participants compared with each city's census.

## Abbreviations

PASTA: Physical Activity through Sustainable Transport Approaches
