# Supplementary material for: Evaluation of Different Recruitment Methods: Longitudinal, Web-Based, Pan-European Physical Activity Through Sustainable Transport Approaches (PASTA) Project
Source: J Med Internet Res. 2019 May 6;21(5):e11492. doi: 10.2196/11492 (PMC6533046; doi:10.2196/11492)
Supplement: Multimedia Appendix 1 [file jmir_v21i5e11492_app1.pdf]

Table h: Demographic characteristic (gender) by recruitment strategy of participants compared with each City Census

	Men (D[a])	Women (D)	chi-squared[b]	*P*
**Antwerp**				
**City Census, 2017 (N = 393,975) %**	**49.4**	**50.6**	-	-
**Total Antwerp (N = 1,444) %**	47.6 (-1.8)	52.4 (1.8)	1.68	**.19**
Work (n = 376) **%**	38.8 (-10.6)	61.2 (10.6)	16.34	< .001
Word of mouth (n = 194) %	50.0 (0.6)	50.0 (-0.6)	0.01	**.92**
Other organizations (n = 289) %	50.9 (1.5)	49.1 (-1.5)	0.20	**.66**
Project outreach activities (n = 132) %	59.1 (9.7)	40.9 (-9.7)	4.59	.03
Media[c] (n = 409) %	48.4 (-1.0)	51.6 (1.0)	0.12	**.73**
News (n = 98) %	55.1 (5.7)	44.9 (-5.7)	1.06	**.30**
Social media (n = 311) %	46.3 (-3.1)	53.7 (3.1)	1.06	**.30**
**Barcelona**				
**City Census, 2016 (N = 1,339,223)**	**46.5**	**53.5**	-	-
**Total Barcelona (N = 1,694) %**	41.1 (-5.4)	58.9 (5.4)	19.48	< .001
Work (n = 198) %	28.3 (-18.2)	71.7 (18.2)	25.76	< .001
Word of mouth (n = 366) %	41.3 (-5.2)	58.7 (5.2)	3.88	.05
Other organizations (n = 105) %	41.0 (-5.5)	59.0 (5.5)	1.10	**.29**
Project outreach activities (n = 650) %	42.5 (-4.0)	57.5 (4.0)	4.15	.04
Media (n = 257) %	47.5 (1.0)	52.5 (-1.0)	0.06	**.81**
News (n = 40) %	50.0 (3.5)	50.0 (-3.5)	0.08	**.78**
Social media (n = 217) %	47.0 (0.5)	53.0 (-0.5)	0.01	**.94**
**London**				
**City Census, 2015 (N = 6,533,667) %**	**49.2**	**50.8**	-	-
**Total London (N = 1,431) %**	41.4 (-7.8)	58.6 (7.8)	34.05	< .001
Work (n= 316) %	37.7 (-11.5)	62.3 (11.5)	16.35	< .001
Word of mouth (n = 140) %	48.6 (-0.6)	51.4 (0.6)	0.004	**.96**
Other organizations (n= 281) %	42.3 (-6.9)	57.7 (6.9)	4.99	.03
Project outreach activities (n = 196) %	44.4 (-4.8)	55.6 (4.8)	1.62	**.20**
Media (n = 351) %	42.2 (-7.0)	57.8 (7.0)	6.65	.01
News (n = 109) %	46.8 (-2.4)	53.2 (2.4)	0.16	**.69**
Social media (n = 242) %	40.1 (-9.1)	59.9 (9.0)	7.67	.01
**Oerebro**				
**City Census, 2016 (N = 112,173)**	**48.8**	**51.2**	-	-
**Total Oerebro (N = 1,395) %**	37.8 (-11.0)	62.2 (11.0)	65.40	< .001
Work (n = 342) %	34.5 (-14.3)	65.5 (14.3)	27.23	< .001
Word of mouth (n = 15) %	60 (11.2)	40 (-11.2)	0.37	**.54**
Other organizations (n = 36) %	50 (1.2)	50 (-1.2)	< 0.001	**1**
Project outreach activities (n = 547) %	35.1 (-13.7)	64.9 (13.7)	40.20	< .001
Media (n = 84) %	34.5 (-14.3)	65.5 (14.3)	6.26	.01
News (n = 51) %	37.3 (-11.5)	62.7 (11.5)	2.27	**.13**
Social media (n = 33) %	30.3 (-18.5)	69.7 (18.5)	3.80	**.05**
Random sampling (n = 360) %	43.9 (-4.9)	56.1 (4.9)	3.24	**.07**
**Rome**				
**City Census, 2011 (N = 2,150,368) %**	**46.0**	**54.0**	-	-
**Total Rome (N = 1,836) %**	61.1 (15.1)	38.9 (-15.1)	168.33	< .001
Work (n = 337) %	54.6 (8.6)	45.4 (-8.6)	9.72	.002
Word of mouth (n = 244) %	57.8 (11.8)	42.2(-11.8)	13.21	< .001
Other organizations (n = 140) %	62.9 (16.9)	37.1 (-16.9)	15.37	< .001
Project outreach activities (n = 158) %	66.5 (20.5)	33.5 (-20.5)	25.83	< .001
Media (n = 780) %	63.3 (17.3)	36.7 (-17.3)	93.76	< .001
News (N = 248) %	57.7 (11.7)	42.3 (-11.7)	13.14	< .001
Social media (N = 89) %	66.0 (20.0)	34.0 (-20.0)	84.77	< .001
Public notice[d] (N = 532) %	74.2 (28.2)	25.8 (-28.2)	27.31	< .001

Vienna				
**City Census, 2016 (N = 1,486,591) %**	**47.8**	**52.2**	-	-
**Total Vienna (N = 1,461) %**	46.4 (-1.4)	53.6 (1.4)	1.11	**.29**
Work (N = 230) %	35.2 (-12.6)	64.8 (12.6)	14.13	< .001
Word of mouth (N = 154) %	39.0 (-8.8)	61.0 (8.8)	4.49	.034
Other organizations (N = 314) %	59.2 (11.4)	40.8 (-11.4)	15.95	< .001
Project outreach activities (N = 327) %	46.5 (-1.3)	53.5 (1.3)	0.18	**.67**
Media (N = 380) %	45.5 (-2.3)	54.5 (2.3)	0.71	**.40**
News (N = 108) %	47.2 (-0.6)	52.8 (0.6)	< 0.001	**.98**
Social media (N = 272) %	44.9 (-2.9)	55.1 (2.9)	0.84	**.36**
**Zurich**				
**City Census, 2015 (N = 343,379)**	**49.5**	**50.5**	-	-
**Total Zurich (N = 1,329) %**	43.2 (-6.3)	56.8 (6.3)	20.68	< .001
Work (N = 487) %	39.4 (-10.1)	60.6 (10.1)	19.26	< .001
Word of mouth (N = 93) %	50.5 (1.0)	49.5 (-1.0)	0.01	**.92**
Other organizations (N = 178) %	42.7 (-6.8)	57.3 (6.8)	3.01	**.08**
Project outreach activities (N = 185) %	43.2 (-6.3)	56.8 (6.3)	2.63	**.10**
Media (N = 372) %	46.8 (-2.7)	53.2 (2.7)	0.98	**.32**
News (N = 134) %	50.7 (1.2)	49.3 (-1.2)	0.04	**.84**
Social media (N = 238) %	44.5 (-5.0)	55.5 (5.0)	2.13	**.14**

[a] Deviation to census data (effect size)

[b] Pearson`s Chi-squared test

[c] Category media combines news and social media into one category

[d] Sample size reached through public notice was only in Rome sufficient big
