# Supplementary material for: Evaluation of Different Recruitment Methods: Longitudinal, Web-Based, Pan-European Physical Activity Through Sustainable Transport Approaches (PASTA) Project
Source: J Med Internet Res. 2019 May 6;21(5):e11492. doi: 10.2196/11492 (PMC6533046; doi:10.2196/11492)
Supplement: Multimedia Appendix 2 [file jmir_v21i5e11492_app2.pdf]

## Appendix

**Table a: Demographic characteristic (age groups) by recruitment strategy of participants of Antwerp compared with City Census Data of Antwerp from 2017**

| **Antwerp** | **20-29 (D[a])** | **30-39 (D)** | **40-49 (D)** | **50-59 (D)** | **60+ (D)** | **chi-squared[b]** | ***P*** |
|---|---|---|---|---|---|---|---|
| **City Census (N = 393,975) %** | **18.7** | **20.2** | **16.9** | **15.4** | **28.7** | - | - |
| **Total Antwerp (N = 1,444) %** | **13.2** (-5.5) | **29.6** (9.4) | **24.4** (7.5) | **21.7** (6.3) | **11.0** **(-17.7)** | 328.77 | < .001 |
| Work (n = 376) % | 13.6 (-5.1) | 33.0 (12.8) | 28.2 (11.3) | 21.0 (5.6) | 4.3 **(-24.4)** | 149.95 | < .001 |
| Word of mouth (n = 194) % | 21.6 (2.9) | 30.4 (10.2) | 19.1 (2.2) | 16.0 (0.6) | 12.9 **(-15.8)** | 28.38 | < .001 |
| Other organizations (n = 289) % | 8.3 (-10.4) | 27.3 (7.1) | 24.6 (7.7) | 23.9 (8.5) | 15.9 **(-12.8)** | 63.91 | < .001 |
| Project outreach activities (n = 132) % | 12.9 (-5.8) | 31.1 (10.9) | 18.2 (1.3) | 29.5 (14.1) | 8.3 **(-20.4)** | 46.33 | < .001 |
| Media[c] (n = 409) % | 12.5 (-6.2) | 26.7 (6.5) | 25.9 (9.0) | 20.5 (5.1) | 14.4 **(-14.3)** | 72.65 | < .001 |
| News (n = 98) % | 8.2 (-10.5) | 24.5 (4.3) | 27.6 (10.7) | 19.4 (4.0) | 20.4 (-8.3) | 16.68 | .002 |
| Social media (n = 311) % | 13.8 (-4.9) | 27.3 (7.1) | 25.4 (8.5) | 20.9 (5.5) | 12.5 **(-16.2)** | 59.49 | < .001 |

[a] Deviation to census data (effect size)
[b] Person´s Chi-squared test
[c] Category media combines news and social media into one category

**Table b: Demographic characteristics (age groups) by recruitment strategy of participants of Barcelona compared with City Census Data of Barcelona from 2016**

| **Barcelona** | **20-29 (D)** | **30-39 (D)** | **40-49 (D)** | **50-59 (D)** | **60+ (D)** | **chi-squared** | ***P*** |
|---|---|---|---|---|---|---|---|
| **City Census (N = 1,339,223) %** | **13.4** | **20.0** | **18.4** | **15.7** | **32.5** | - | - |
| **Total Barcelona (N = 1,694) %** | 30.0 (16.6) | 32.1 (12.1) | 19.4 (1.0) | 12.6 (-3.1) | 5.9 **(-26.6)** | 851.36 | < .001 |
| Work (n = 198) % | 23.7 (10.3) | 49.0 (29.0) | 18.7 (0.3) | 7.1 (-8.6) | 1.5 **(-31.0)** | 166.81 | < .001 |
| Word of mouth (n = 366) % | 33.3 (19.9) | 32.0 (12.0) | 19.4 (1.0) | 10.9 (-4.8) | 4.4 **(-28.1)** | 228.97 | < .001 |
| Other organizations (n = 105) % | 32.4 (19.0) | 25.7 (5.7) | 18.1 (-0.3) | 15.2 (-0.5) | 8.6 **(-23.9)** | 48.386 | < .001 |
| Project outreach activities (n = 650) % | 30.2 (16.8) | 27.7 (7.7) | 17.8 (-0.6) | 15.4 (-0.3) | 8.9 **(-23.6)** | 266.18 | < .001 |
| Media (n = 257) % | 23.0 (9.6) | 38.9 (18.9) | 23.7 (5.3) | 10.9 (-4.8) | 3.5 **(-29.0)** | 137.65 | < .001 |
| News (n = 40) % | 22.5 (9.1) | 37.5 (17.5) | 25.0 (6.6) | 7.5 (-8.2) | 7.5 **(-25.0)** | 18.94 | < .001 |
| Social media (n = 217) % | 23.0 (9.6) | 39.2 (19.2) | 23.5 (5.1) | 11.5 (-4.2) | 2.8 **(-29.7)** | 119.39 | < .001 |

**Table c: Demographic characteristics (age groups) by recruitment strategy of participants of London compared with City Census Data of London from 2015**

| London | 20-29 (D) | 30-39 (D) | 40-49 (D) | 50-59 (D) | 60+ (D) | chi-squared | *P* |
|---|---|---|---|---|---|---|---|
| **City Census (N = 6,533,667) %** | **21.5** | **24.5** | **18.6** | **14.8** | **20.6** | - | - |
| **Total London (N = 1,431) %** | 22.9 (1.4) | 30.6 (6.1) | 20.6 (2.0) | 16.2 (1.4) | 9.7 **(-10.9)** | 110.69 | < .001 |
| Work (n= 316) % | 23.1 (1.6) | 33.9 (9.4) | 21.5 (2.9) | 16.1 (1.3) | 5.4 **(-15.2)** | 49.15 | < .001 |
| Word of mouth (n = 140) % | 32.9 (11.4) | 39.3 (14.8) | 17.9 (-0.7) | 5.7 (-9.1) | 4.3 **(-16.3)** | 46.84 | < .001 |
| Other organizations (n= 281) % | 13.5 (-8.0) | 28.5 (4.0) | 20.3 (1.7) | 24.2 (9.4) | 13.5 (-7.1) | 34.38 | < .001 |
| Project outreach activities (n = 196) % | 33.2 (11.7) | 24.0 (-0.5) | 15.3 (-3.3) | 14.3 (-0.5) | 13.3 (-7.3) | 18.64 | < .001 |
| Media (n = 351) % | 18.5 (-3.0) | 28.2 (3.7) | 25.4 (6.8) | 15.4 (0.6) | 12.5 **(-8.1)** | 23.21 | < .001 |
| News (n = 109) % | 12.8 (-8.7) | 31.2 (6.7) | 25.7 (7.1) | 16.5 (1.7) | 13.8 (-6.8) | 11.46 | .02 |
| Social media (n = 242) % | 21.1 (-0.4) | 26.9 (2.4) | 25.2 (6.6) | 14.9 (0.1) | 12.0 **(-8.6)** | 14.95 | .005 |

**Table d: Demographic characteristics (age groups) by recruitment strategy of participants of Oerebro compared with City Census Data of Oerebro from 2016**

| Oerebro | 20-29 (D) | 30-39 (D) | 40-49 (D) | 50-59 (D) | 60+ (D) | chi-squared | *P* |
|---|---|---|---|---|---|---|---|
| **City Census (N = 112,173) %** | **21.2** | **17.0** | **16.6** | **14.9** | **30.4** | - | - |
| **Total Oerebro (N = 1,395) %** | 18.6 (-2.6) | 20.4 (3.4) | 19.5 (2.9) | 19.9 (5.0) | 21.6 **(-8.8)** | 77.51 | < .001 |
| Work (n = 342) % | 9.9 (-11.3) | 21.9 (4.9) | 22.8 (6.2) | 27.8 (12.9) | 17.5 **(-12.9)** | 89.43 | < .001 |
| Word of mouth (n = 15) % | 6.7 (-14.5) | 13.3 (-3.7) | 26,7 (10.1) | 33.3 (18.4) | 20.0 (-10.4) | 6.50 | **.17**[a] |
| Other organizations (n =  36) % | 25.0 (3.8) | 16.7 (-0.3) | 38.9 (22.3) | 13.9 (-1.0) | 5.6 **(-24.8)** | 18.32 | .001 |
| Project outreach activities (n = 547) % | 27.4 (6.2) | 19,7 (2.7) | 18.3 (1.7) | 15.4 (0.5) | 19.2 **(-11.2)** | 35.94 | < .001 |
| Media (n = 84) % | 4.8 (-16.4) | 13.1 (-3.9) | 15.5 (-1.1) | 26.2 (11.3) | 40.5 (10.1) | 21.49 | < .001 |
| News (n = 51) % | 3.9 (-17.3) | 7.8 (-9.2) | 19.6 (3.0) | 27.5 (12.6) | 41.2 (10.8) | 17.29 | .002 |
| Social media (n = 33 ) % | 6.1 (-15.1) | 21.2 (4.2) | 9.1 (-7.5) | 24.2 (9.3) | 39.4 (9.0) | 7.85 | **.10**[a] |
| Random sampling (n = 360) % | 16.1 (-5.1) | 22.2 (5.2) | 17.5 (0.9) | 17.5 (2.6) | 26.7 (-3.7) | 13.54 | .009 |

[a] Small sample size; chi-squared approximation might be incorrect

**Table e: Demographic characteristics (age groups) by recruitment strategy of participants of Rome compared with City Census Data of Rome from 2011**

| Rome | 20-29 (D) | 30-39 (D) | 40-49 (D) | 50-59 (D) | 60+ (D) | chi-squared | *P* |
|---|---|---|---|---|---|---|---|
| **City Census (N = 2,150,368) %** | **11.7** | **17.0** | **20.8** | **16.4** | **34.1** | - | - |
| **Total Rome (N = 1,836) %** | 19.8 (8.1) | 29.8 (12.8) | 25.5 (4.7) | 20.3 (3.9) | 4.5 **(-29.6)** | 787.67 | < .001 |
| Work (n = 337) % | 11.0 (-0.7) | 27.0 (10.0) | 27.6 (6.8) | 27.0 (10.6) | 7.4 **(-26.7)** | 120.87 | < .001 |
| Word of mouth (n = 244) % | 21.3 (9.6) | 37.7 (20.7) | 22.1 (1.3) | 16.4 (0.0) | 2.5 **(-31.6)** | 152.48 | < .001 |
| Other organizations (n =  140) % | 24.3 (12.6) | 22.1 (5.1) | 26.4 (5.6) | 23.6 (7.2) | 3.6 **(-30.5)** | 65.92 | < .001 |
| Project outreach activities (n = 158) % | 26.6 (14.9) | 19.6 (2.6) | 24.7 (3.9) | 25.9 (9.5) | 3.2 **(-30.9)** | 84.80 | < .001 |

| | | | | | | | |
|---|---|---|---|---|---|---|---|
| Media (n = 780) % | 18.5 (6.8) | 35.0 (18.0) | 26.2 (5.4) | 16.2 (-0.2) | 4.2 **(-29.9)** | 393.65 | < .001 |
| News (N = 248) % | 19.4 (7.7) | 28.6 (11.6) | 27.8 (7.0) | 19.0 (2.6) | 5.2 **(-28.9)** | 99.57 | < .001 |
| Social media (N = 89) % | 18.0 (6.3) | 38.0 (21.0) | 25.4 (4.6) | 14.8 (-1.6) | 3.8 **(-30.3)** | 305.37 | < .001 |
| Public notice[a] (N = 532) % | 49.4 (37.7) | 11.2 (5.8) | 15.7 (-5.1) | 15.7 (-0.7) | 7.9 **(-26.2)** | 129.13 | < .001 |

[a] Sample size reached through public notice was only in Rome sufficient big

**Table f: Demographic characteristics (age groups) by recruitment strategy of participants of Vienna compared with City Census Data of Vienna from 2016**

| **Vienna** | **20-29 (D)** | **30-39 (D)** | **40-49 (D)** | **50-59 (D)** | **60+ (D)** | **chi-squared** | ***P*** |
|---|---|---|---|---|---|---|---|
| **City Census (N = 1,486,591) %** | **19.6** | **19.0** | **18.1** | **16.6** | **26.7** | - | - |
| **Total Vienna (N = 1,461) %** | 27.3 (7.7) | 27.7 (8.7) | 19.8 (1.7) | 16.9 (0.3) | 8.4 **(-18.3)** | 288.11 | < .001 |
| Work (N = 230) % | 21.3 (1.7) | 34.8 (15.8) | 27.4 (9.3) | 14.8 (-1.8) | 1.7 **(-25.0)** | 95.47 | < .001 |
| Word of mouth (N = 154) % | 31.2 (11.6) | 40.9 (21.9) | 13.0 (-5.1) | 10.4 (-6.2) | 4.5 **(-22.2)** | 83.64 | < .001 |
| Other organizations (N = 314) % | 24.2 (4.6) | 27.1 (8.1) | 17.8 (-0.3) | 19.7 (3.1) | 11.1 **(-15.6)** | 44.38 | < .001 |
| Project outreach activities (N = 327) % | 29.4 (9.8) | 21.7 (2.7) | 21.4 (3.3) | 18.0 (1.4) | 9.5 **(-17.2)** | 55.77 | < .001 |
| Media (N = 380) % | 28.9 (9.3) | 24.5 (5.5) | 19.2 (1.1) | 17.9 (1.3) | 9.5 **(-17.2)** | 65.75 | < .001 |
| News (N = 108) % | 26.9 (7.3) | 18.5 (0.5) | 16.7 (-1.4) | 19.4 (2.8) | 18.5 **(-8.2)** | 6.25 | **.18** |
| Social media (N = 272) % | 29.8 (10.2) | 26.8 (7.8) | 20.2 (2.1) | 17.3 (0.7) | 5.9 **(-20.8)** | 68.04 | < .001 |

**Table g: Demographic characteristics (age groups) by recruitment strategy of participants of Zurich compared with City Census Data of Zurich from 2015**

| **Zurich** | **20-29 (D)** | **30-39 (D)** | **40-49 (D)** | **50-59 (D)** | **60+ (D)** | **chi-squared** | ***P*** |
|---|---|---|---|---|---|---|---|
| **City Census (N = 343,379) %** | **20.1** | **25.1** | **17.9** | **13.8** | **23.1** | - | - |
| **Total Zurich (N = 1,329) %** | 21.7 (1.6) | 28.6 (3.5) | 21.7 (3.8) | 19.7 (5.9) | 8.2 **(-14.9)** | 179.7 | < .001 |
| Work (N = 487) % | 17.9 (-2.2) | 30.2 (5.1) | 23.6 (5.7) | 22.6 (8.8) | 5.7 **(-17.4)** | 105.41 | < .001 |
| Word of mouth (N = 93) % | 24.7 (4.6) | 29.0 (3.9) | 21.5 (3.6) | 11.8 (-2.0) | 12.9 **(-10.2)** | 6.70 | **.15** |
| Other organizations (N = 178) % | 13.5 (-6.6) | 28.1 (3.0) | 24.7 (6.8) | 27.5 (13.7) | 6.2 **(-16.9)** | 55.26 | < .001 |
| Project outreach activities (N = 185) % | 28.1 (8.0) | 27.0 (1.9) | 11.4 (-6.5) | 17.3 (3.5) | 16.2 (-6.9) | 16.04 | < .001 |
| Media (N = 372) % | 26.3 (6.2) | 27.4 (2.3) | 23.7 (5.8) | 15.6 (1.8) | 7.0 **(-16.1)** | 57.50 | < .001 |
| News (N = 134) % | 20.9 (0.8) | 21.6 (-3.5) | 27.6 (9.7) | 17.2 (3.4) | 12.7 **(-0.4)** | 15.07 | .005 |
| Social media (N = 238) % | 29.4 (9.3) | 30.7 (5.6) | 21.4 (3.5) | 14.7 (0.9) | 3.8 **(-19.3)** | 53.48 | < .001 |
